# Chronic Pain in Children and Adolescents: Diagnosis and Treatment of Primary Pain Disorders in Head, Abdomen, Muscles and Joints

**DOI:** 10.3390/children3040042

**Published:** 2016-12-10

**Authors:** Stefan J. Friedrichsdorf, James Giordano, Kavita Desai Dakoji, Andrew Warmuth, Cyndee Daughtry, Craig A. Schulz

**Affiliations:** 1 Children’s Hospitals and Clinics of Minnesota, Minneapolis, MN 55404, USA; Kavita.DesaiDakoji@childrensMN.org (K.D.D.); Andrew.Warmuth@childrensMN.org (A.W.); Cyndee.Daughtry@childrensMN.org (C.D.); Craig.Schulz@childrensMN.org (C.S.); 2 Department of Pediatrics, University of Minnesota Medical School, Minneapolis, MN 55455, USA; 3 Georgetown University Medical Center, Washington, DC 20057, USA; james.giordano@georgetown.edu; 4 Center for Spirituality & Healing, University of Minnesota, Minneapolis, MN 55455, USA

**Keywords:** chronic pain, interdisciplinary treatment, children, adolescents, biopsychosocial, primary pain disorder, pediatric pain clinic

## Abstract

Primary pain disorders (formerly “functional pain syndromes”) are common, under-diagnosed and under-treated in children and teenagers. This manuscript reviews key aspects which support understanding the development of pediatric chronic pain, points to the current pediatric chronic pain terminology, addresses effective treatment strategies, and discusses the evidence-based use of pharmacology. Common symptoms of an underlying pain vulnerability present in the three most common chronic pain disorders in pediatrics: primary headaches, centrally mediated abdominal pain syndromes, and/or chronic/recurrent musculoskeletal and joint pain. A significant number of children with repeated acute nociceptive pain episodes develop chronic pain in addition to or as a result of their underlying medical condition “chronic-on-acute pain.” We provide description of the structure and process of our interdisciplinary, rehabilitative pain clinic in Minneapolis, Minnesota, USA with accompanying data in the treatment of chronic pain symptoms that persist beyond the expected time of healing. An interdisciplinary approach combining (1) rehabilitation; (2) integrative medicine/active mind-body techniques; (3) psychology; and (4) normalizing daily school attendance, sports, social life and sleep will be presented. As a result of restored function, pain improves and commonly resolves. Opioids are not indicated for primary pain disorders, and other medications, with few exceptions, are usually not first-line therapy.

## 1. Introduction

Pediatric chronic pain is a significant problem with conservative estimates that posit 20% to 35% of children and adolescents affected by it worldwide [1,2,3]. Pain experienced in children’s hospitals is known to be common, under-recognized, and under-treated, with more than 10% of hospitalized children showing features of chronic pain [4,5,6,7]. Although the majority of children reporting chronic pain are not greatly disabled by it [8], about 3% of pediatric chronic pain patients require intensive rehabilitation [9]. The total costs to society incurred by care for children and adolescents with moderate to severe chronic pain has been extrapolated to $19.5 billion annually in the USA [10]. For a recent systematic review of the etiology of chronic and recurrent pediatric pain not associated with disease, see King et al., 2011 [2].

The underlying pathophysiology of pain in children includes acute nociceptive pain (i.e., pain arising from the activation of peripheral nerve endings, including somatic and visceral pain), neuropathic pain (i.e., resulting from injury to, or dysfunction of, the somatosensory system), psycho-social-spiritual-emotional pain, total pain and/or the topic of this paper, chronic pain. Pain may originate from one, but more commonly involves a combination of these pathophysiologies. Commonly accepted definitions of chronic pain describe pain that persists for three months; however, the Rome IV criteria for functional abdominal pain disorders, for instance, typically require symptoms to be present and persist for at least two months [11]. Like many pediatric pain programs, we define chronic pain not necessarily by using arbitrary temporal parameters, but rather employ a more functional definition such as “pain that extends beyond the expected period of healing” and “hence lacks the acute warning function of physiological nociception” [12,13]. Simply put, pain lasting two months and 29 days does not necessarily change from “acute” to “chronic” once it merely extends to a period of three months in duration.

The 2012 American Pain Society Position Statement, “Assessment and Management of Children with Chronic Pain”, indicates that chronic pain in children is the result of a dynamic integration of biological processes, psychological factors, and sociocultural variables, considered within a developmental trajectory [14]. Chronic pain includes persistent (ongoing) and recurrent (episodic) pain in children with underlying health conditions (e.g., inflammatory bowel disease, sickle cell disease, rheumatoid arthritis), and pain that is the disorder itself (e.g., primary headaches, centrally mediated abdominal pain syndrome, musculoskeletal pain, complex regional pain syndrome), with a significant number of children experiencing both entities, i.e., “chronic-on-acute” pain. Chronic pain affects the entire nervous system, and the term central sensitization, which is an increased central neural responsiveness to painful and non-painful stimuli, has been used to describe any central nervous system dysfunction or pathology that may contribute to the development or maintenance of many types of chronic pain [14,15].

Chronic childhood and adolescent pain is not only an issue of importance for the clinical care of pediatric patients, but also is a condition that exerts considerable bearing upon the medical, social and economic sectors. We believe that an improved understanding, definition and approach to treating chronic childhood and adolescent pain represent important steps toward both optimizing the clinical care of pediatric pain patients, and in this way, diminishing the overall negative impact of chronic pain on patients, medicine and society. In this light, and as an introduction to the Special Issue “Chronic and Recurrent Pain” in *Children* edited by L.S. Walker and C.L. von Baeyer [16], the aim of this paper is to highlight important key aspects in support of a fortified understanding of the development and expression of chronic pain in children and adolescents, such as pain catastrophizing and fear of pain. In addition, we aim to review the current pediatric chronic pain terminology and provide a brief description of the approach to assessing and treating chronic pain as practiced in our interdisciplinary pediatric pain clinic. 

## 2. Children with Chronic Pain

### 2.1. Trajectory

Untreated chronic pain in children incurs a high risk for the subsequent development of pain and psychological disorders later in life. Seventeen percent of adult chronic pain patients reported a history of chronic pain in childhood or adolescence, with close to 80% indicating that the pain in childhood continued and persisted until adulthood [17]. In the USA, adults with chronic pain have a lower household income and higher risk of unemployment [18]. Studies of two birth cohorts from 1946 and 1958 showed that children with persistent abdominal pain and headaches go on to suffer more physical symptoms, anxiety and depression in adult life than healthy children [19,20,21,22]. A prospective study by Mulvaney and colleagues of 132 children with abdominal pain indicated long-term, high level risk of (adult) symptoms and impairment for a cluster of patients who did not have the most severe pain, but who had significantly more anxiety, depression, lower perceived self-worth, and more negative life events at baseline [23].

In another three-year prospective cohort study [24] involving 1336 children and teens in pain aged 11–14 years, Dunn et al. showed that 44% displayed a developmental trajectory for increased pain disorders and conditions, primarily presenting as headaches, back pain, abdominal pain and facial pain, and 12% presented with persistent pain. Individuals at highest risk to develop persistent pain were predominantly female, demonstrated the highest level of somatization and depression at both the start and end of the study period, and were least likely to be satisfied with their life. As well, research has shown that extra-intestinal somatic and depressive symptoms at the initial pediatric evaluation for functional abdominal pain were significant predictors of functional gastrointestinal disorders in adulthood [25]. Pediatric patients with functional abdominal pain exhibit long-term vulnerability to anxiety that begins in childhood and persists into late adolescence and early adulthood, even if abdominal pain resolves [26]. The National Longitudinal Study of Adolescent to Adult Health included more than 14,000 study participants and has shown that chronic pain in adolescence is associated with higher rates of mental health disorders reported in adulthood with anxiety (21.1% vs. 12.4% pain-free adolescents) and depressive disorders (24.5% vs. 14.1%) being most common [27]. Van Tilburg and colleagues analyzed data from a longitudinal study of a nationally representative sample of 9970 adolescents in the USA to show that adolescents with chronic pain and depression are at increased risk for both suicidal ideation and suicide attempt [28].

### 2.2. Pain Catastrophizing and Fear of Pain

The fear avoidance model of pain with an emphasis on the maladaptive behaviors that lead to activity avoidance has guided pediatric chronic pain research and clinical practice [29]. Certainly most, if not all, people seek to avoid pain. Fear of pain and subsequent exacerbation of its effects is common in both adults and children. Catastrophizing (or “Awfulizing”) is a key mechanism by which the experience of pain can be exacerbated in children and adolescents. Catastrophizing represents a set of negative cognitive/emotional processes that include magnification (amplification of the significance of pain), rumination (anxious preoccupation with pain) and pessimism about pain sensations and feelings of helplessness when in pain. There is a significant body of research showing a correlation of children’s pain behaviors and their (as well as their parents’) extent of catastrophizing [30,31,32]. Pain reduction through distraction is delayed by catastrophizing [33], is associated with greater pain in children when either child or parent displays high pain catastrophizing [34]. Negative pain memory bias is advanced by parental (more than children’s) pain catastrophizing and, in addition, expectations as well as cognitions of parents influence child pain memory formation [35].

Both physical and psychological dysfunction in pediatric chronic pain is modulated and upregulated by fear of pain [36]. Diminishing the fear of pain strongly correlates with positive functional outcomes in children with primary pain disorders [36]. Further, there is a robust positive association between pain-related fear and disability [37]. Consistent with the fear-avoidance model of chronic pain, these findings suggest that pain-related fear may be an important therapeutic target for approaches aimed at reducing pain-related disability.

### 2.3. Time for a New Name: “Primary Pain Disorder”

Many different chronic and recurrent pain syndromes in both adult and pediatric populations are now considered to be manifestations of an underlying vulnerability or pain spectrum condition, rather than being viewed as separate disorders [38]. Considerable evidence, especially from twin studies, points to a role of shared biological sensitivity, “pain vulnerability,” “pain sensitivity,” or “central sensitivity syndrome” [38,39,40,41,42]. Thus, conditions such as primary headaches (including tension headaches), centrally mediated abdominal pain syndromes, localized or widespread musculoskeletal pain (including “pain amplification syndrome,” “non-cardiac chest pain,” “costochondritis,” “temporomandibular joint disorder,” “juvenile fibromyalgia,” etc.) are no longer regarded as separate entities with differing underlying pathophysiologies in need of different treatments. Instead, these conditions are now generally regarded as pain manifestations of the same underlying condition that present at varied locations.

The term primary pain disorder (formerly: functional pain syndrome) was first employed by Schechter in 2014 [43]. It describes a chronic pain disorder that cannot be explained by appropriate medical assessment(s) in terms of conventionally defined medical disease, as based on biochemical or structural abnormalities. A primary pain disorder is associated with significant disruption of everyday life and often leads to incapacitation. This disorder is not typically responsive to unimodal medical therapy, and attempts at such management can consume significant time, medical resources and finances. Persistent treatment failures can lead to pejorative implications such as patients perceiving that their pain is not organic and therefore not real or serious, and/or stigmatization (i.e., patients’ symptoms may be characterized as fictitious or as evidence of malingering). 

Toward advancing an improved understanding of primary pain disorder and its relation to chronic pain in children and adolescents, the following section presents a review of the three most prevalent symptoms, manifestations, and locations of a primary pain disorder (headaches, abdominal pain, and/or musculoskeletal pain).

## 3. Pain Manifestations and Locations of a Primary Pain Disorder

### 3.1. Primary Headache: Tension Headaches and Migraines

Traditionally, headaches were classified using a bimodal construct: tension headaches (infrequent episodic, frequent episodic, or chronic) versus migraines (with or without aura) [44]. In 2013 the International Headache Society (IHS) [45] classified “primary headaches” as migraines, tension-type headaches, trigeminal autonomic cephalalgias and “secondary headaches,” e.g., a headache attributed to trauma or injury to the head, cranial or cervical vascular disorders, etc. Pediatric data supports the notion that childhood headaches are a continuum, rather than discrete entities [46,47]. Recent findings [48] suggest that “migraine” and “tension-type” headaches might not be separate diagnostic/disease entities, but rather represent a severity continuum for young adults 18–24 years of age who experience chronic headaches more than 3–4 days per week. Two cross-sectional studies [48] with more than 3400 headache patients revealed that those who are experiencing headaches at a younger age (18–24 years old) and with higher frequency (more than 15 headache days/month) displayed a unimodal distribution, suggesting the dimensional construct of a primary headache. Conversely, patients who experienced a lower frequency of headaches and were of older age showed bimodal headache distributions, which could be differentiated into “migraine” versus “tension-type” headache. In addition, pressure pain thresholds over the temporalis, masseter, and frontalis muscles in patients with headaches do not exhibit strong differences between migraine and tension headaches [49]. In other words, for pediatric patients who are headache-free most of the time and (only) experience episodic headaches, the “bimodal” classification of “tension-type” versus “migraines” continues to be clinically appropriate, as migraines (as opposed to tension-type) might be alleviated by a medication regimen [50,51,52] (see Section 5). On the other hand, for patients who present with chronic, daily headaches (or headaches that occur and persist more than 50% of the time) separation into “tension-type headaches” and “migraines” appears to be a less helpful distinction. For clinical signs that may require further workup of headache, see Table 1.

Medication overuse headaches (MOH) are not uncommon among pediatric patients with chronic headaches and usually occur when patients are taking analgesics (including acetaminophen, non-steroidal anti-inflammatory drugs (NSAIDs), or opioids), triptans, and/or ergotamine for more than 10–15 days/month (depending on medication class) [53,54,55]. Effective treatment requires a rapid discontinuation of medication [56]. In a case study of children and adolescents with daily or almost-daily headache concomitant with daily or almost-daily analgesic intake, Hering-Hanit et al. reported that a successful wean from analgesics was achieved without hospitalization or significant interference with daily life, and resulted in complete cessation of chronic daily headache in 25 of 26 patients [57].

### 3.2. Centrally Mediated Abdominal Pain Syndrome

In the 2016 revision of the Rome Criteria (now in its 4th iteration), the term “functional abdominal pain” has been replaced by “centrally mediated abdominal pain syndrome” (CAPS), which occurs as a result of central sensitization with disinhibition of pain signals, rather than increased peripheral afferent excitability [58]. For example, when compared to healthy peers, pre-adolescent girls with irritable bowel syndrome display impaired endogenous inhibition of somatic pain [59]. Adolescents with irritable bowel syndrome (IBS) symptoms are also more likely to experience widespread hyperalgesia [60]. CAPS in childhood and adolescence increases the risk for chronic primary pain disorder (abdominal pain, migraine/tension headache, and chronic musculoskeletal pain) in adulthood [61,62], and women with a pediatric history of functional abdominal pain display long-term vulnerability to pain [63]. For clinical warning signs that may require further workup of abdominal pain, see Table 2.

### 3.3. Musculoskeletal and Joint Pain

A systematic review by King et al. [2] reported that back pain is common in children and adolescents, with one-month prevalence rates ranging from 18% to 24% in samples of English and Swedish children [64,65]. Weekly or “at least weekly” back pain was reported in 9%–25% of patients [3,64,66].

Stanford et al. have shown that parent- and youth-reported anxiety/depression were predictive of start and end points of back pain trajectories [3]. Four studies reported a 9%–39% prevalence of musculoskeletal and/or limb pain in 3842 children and adolescents, depending on time period of reporting [67,68,69,70]. The relation between musculoskeletal/limb pain and athletic participation confounds findings in this area, as many participants reported that their pain was the result of a sports injury. The majority of the studies reported that musculoskeletal pain is more common in girls than in boys [2].

Diagnostic criteria for “fibromyalgia” are not validated in children and teenagers, and the term “widespread musculoskeletal pain” is more commonly used instead. For clinical warning signs that may require further workup in children presenting with musculoskeletal and/or joint pain, see Table 3.

### 3.4. Orthostatic Dysfunction as Part of the Chronic Pain Picture: From “POTS” and “Autonomic Dysfunction”, to “Chronic Lyme Disease”

During initial intake, more than 50% of our chronic pain patients reveal in their medical history that they experienced episodes of enigmatic symptoms of dizziness, fatigue, low energy, blurry vision, “blacking out,” and/or tachycardia, which we usually interpret as a result of deconditioning; these symptoms are frequently exacerbated by anxiety. We nearly always see this as a result of fear of pain and deconditioning, and not as a unifying diagnosis of a medical condition. 

In short, like the vast majority of pediatric pain centers, we do not support the notion that “post-orthostatic tachycardia syndrome” (POTS), “autonomic dysfunction,” and/or “chronic Lyme disease” (i.e., diagnosis of persistent infection with *Borrelia burgdorferi* despite negative titers calling for long-term antibiotic treatment) [71] are either the underlying pathophysiology or a significant contributor of a primary pain disorder. In fact, in nearly all of our patients, the clinical symptoms of orthostatic dysfunction and other co-existing, enigmatic symptoms (following a reasonable negative workup) disappear during our rehabilitative pain program.

### 3.5. Conversion Disorder

An estimated 5%–10% of patients referred to our pain clinic display clinical signs of a conversion disorder, either singularly or more commonly in conjunction with a primary pain disorder. Conversion disorder is a condition, in which the patient may present with numbness, paralysis, blindness, inability to speak, and/or other neurologic symptoms that cannot be explained by medical evaluation and for which diagnostic testing does not reveal any physical cause [72,73]. Often, the patient presents with a debilitating symptom that begins suddenly, has a history of a psychological problem(s) that gets better after the symptom appears, and shows lack of concern that would usually occur with symptoms of such severity (e.g., a 12-year-old boy seen in our clinic who was not concerned by the fact that he could not bear any weight on his limb and had been in a wheelchair for eight months). 

### 3.6. Children with Acute and Chronic Pain: Co-Existing Pain Entities Requiring Advanced Treatment Strategies

Primary pain disorders also co-exist, or can even be triggered by underlying organic disease, and pain symptoms do not necessarily represent inadequate treatment, flare-up, or recurrence. As previously mentioned, approximately 5% of children and teenagers in the general population have significant pain-related dysfunction [2]. As a result, we can expect that at least the same percentage of children with recurrent painful episodes, such as that occurring in sickle cell disease, inflammatory bowel disease, rheumatoid arthritis, congenital heart disease, or cancer, will display chronic pain features in addition (i.e., co-existance) to their underlying somatic pain episodes, and that pain is not necessarily caused by the organic disease itself.

The realization that a significant number of children with recurrent organic painful episodes whose pain proves to be complicated to treat, have what is known as “chronic-on-acute” disease, which may be potentially complicated even further by neuropathic and/or psycho-social-spiritual pain. Such pain is important, and we believe it has beneficial, clinical implications. Namely, it reduces over-investigation of the underlying organic disease, and over-treatment of pain (e.g., inappropriate long-term opioid administration). Clearly, repeated acute pain episodes increase the risk of significant pain-related dysfunction. For instance, chronic pain is significantly higher in survivors of childhood cancer than in their healthy siblings [74]. Chronic post-surgical pain (CPSP), potentially a transition from acute to chronic pain, has recently been described to occur in 12%–22% of children [75,76,77], and is possibly associated with parental catastrophizing [78].

In practical terms, for treatment of acute tissue injuries (such as new vaso-occlusive sickle cell disease or a flare-up of Crohn’s disease with bloody diarrhea), short-term administration of opioids titrated to effect are in fact usually required for adequate analgesia as part of an advanced multimodal analgesic strategy [79,80]. However, as discussed below (in Section 5.3), in patients with chronic daily sickle-cell pain or chronic daily abdominal pain due to Crohn’s disease that is in clinical remission, long-term daily administration of opioids would not be indicated.

## 4. Interdisciplinary Rehabilitative Pediatric Pain Program: Our Approach

Since 2006, our interdisciplinary rehabilitative pain clinic at Children’s Hospitals and Clinics of Minnesota in Minneapolis, MN, USA, has been dedicated to the care of children and teenagers in pain. A multidisciplinary bio-psycho-social rehabilitation and functional restoration approach has been shown to effectively and efficiently improve function both in adults [81] and children/teenagers [82,83,84,85,86]. However, it remains unclear how long we can wait to send a child to a pain program, and it is also unknown at what point clinical deterioration begins. Adult meta-data show that wait times for chronic pain treatment of six months or longer are medically unacceptable and result in significant decreases in both health-related quality of life and psychological well-being [87]. Two-thirds of USA pediatricians felt it was not their primary responsibility to treat chronic pain [88]. The Pediatric Pain Screening Tool (PPST) was recently validated to assist clinicians stratifying their pediatric pain patients to appropriate interventions [89].

The three most commonly seen manifestations of a primary pain disorder in our pain clinic are (1) primary headache (tension headaches/migraines); (2) centrally mediated abdominal pain syndrome; and (3) chronic and recurrent musculoskeletal/joint pain, with the majority of patients having at least two of those symptoms. The frequency of these conditions is followed closely by the prevalence of Complex regional pain syndrome (CRPS) Type 1, with 11% of our patients presenting with this condition (See Table 6).

Consistent with the treatment approach of the majority of pediatric pain clinics in the USA and Canada [90], our clinic offers an interdisciplinary rehabilitative approach aiming at returning to normal function. 

### 4.1. From Clinic Intake to the Exit Interview

In our clinic, children whose daily functioning is disrupted due to pain (e.g., are missing school, displaying poor sleep, withdrawing from social life and/or having been assessed with anxiety or depression) will be offered a 90-minute multidisciplinary intake (in the same room at the same time) with the patient/parents and a physical therapist, psychologist, social worker/family therapist and a pain physician or advanced nurse practitioner. Following this, the patient undergoes three individual evaluations: clinical examination, a physical therapy evaluation, and a psychological evaluation. Parents/caregivers meet separately with the social worker/family therapist for an evaluation of family factors that may impact the child’s pain, including beliefs about pain and response to pain. Afterwards, the clinical team meets to discuss findings and suggest recommendations, while the family is given a short rest period/hiatus. Following the clinical team meeting, either the MD or NP meets with the patient and his or her family for a 60-minute exit interview to present and explain recommendations and treatment plan.

We found it most effective for a child with a primary pain disorder to return to normal function first, and as a result of such functional reintegration, to then subsequently focus upon decreasing pain subsequently. During the exit interview we reveal that we believe the child’s pain to be “real,” and establish the expectation that as a first step we work toward “life gets back to normal,” and then as a result—“the pain gets better”—and not the other way around. For patients with a primary pain disorder, we set the expectation of becoming pain-free or mostly pain-free. We have found that this positive expectation represents something of a self-fulfilling prophecy, and data support that expectations predict chronic pain treatment outcomes [91]. In our clinic, only 16% of children at intake reported that they believed they could ever be pain-free. Upon exit interview at the end of the first visit, which represents the first formal intervention, 92% of children expressed the belief that they could, in fact, become pain-free.

We put great importance on demystifying the problem: pain has lost its warning signal, and using the affected body part does not result in greater harm. During the exit interview, we explain in age-appropriate language how the pain “is real,” but “has lost its warning function”. Using drawings on a flip-chart and age-appropriate examples, we then go into greater detail about the concept of pain transmission and important modalities to down-modulate pain (so-called “OFF-Switch” modulation) through activation of descending inhibiting pathways (DIP) from the “control-center” periaqueductal grey (PAG), which are engaged by (1) physical therapy/exercise and (2) integrative medicine/distraction. We also discuss up-regulation (so called “ON-switch” activation induced by the prefrontal cortex which worsens pain through stress, negative mood, anxiety, depression, school absenteeism, social withdrawal, insomnia, etc.) and which is mitigated by (3) psychological counseling; and (4) normalizing activities of daily life. We then discuss the need to target both shutting off the “ON-switch” and turning on the “OFF-switch” at the same time, explaining why all four modalities are offered concurrently in our clinic, and why these modalities might not have worked in the past, if and when administered separately. 

### 4.2. Rehabilitative Pain Program Modalities

In order to enroll in our rehabilitative pain program, we expect children and their parents to participate in all (i.e., not just some) of the following five modalities.

#### 4.2.1. Physical Therapy

Physical therapy and exercise are key modalities in the treatment of pediatric patients with primary pain disorder and/or CRPS [82,84,85,86,92,93,94,95]. Adolescents with chronic pain usually have a lower physical activity level [96] and physical activity has been shown to reduce the risk for depression in female adolescents [97]. In children and adolescents participating in a rehabilitative pain program, the rate of improvement in function was significantly more rapid than the decrease in pain [92].

In our clinic, a physical therapist (PT) facilitates restoration of movement and the reduction of pain. PTs develop an individualized treatment plan that is based on each patient’s functional goals (e.g., returning to a sport or age-appropriate play activity). PTs who work with children with chronic pain utilize traditional therapeutic techniques (e.g., normalizing lost range of motion, strength, balance, etc.) along with more pain-specific interventions (e.g., graded motor imagery [98], pain-physiology education, etc.). The majority of chronic pain patients entering our clinic are found to be de-conditioned upon physical therapy evaluation. Clinically, many of our chronic pain patients place a high value on athletics, with the majority of patients participating in some form of sport. If pain disrupts physical activity, it likely also impacts patients’ coping strategies as well as their socialization. Reasons we assert to be important to refer pediatric/adolescent pain patients to physical therapy are provided in Table 4.

The treatment goal we establish for and with our patients is to first return to function, and then to secondarily attempt to decrease pain as a result of this functional restoration.

The initial assessment goals include determining if any neuro-musculoskeletal factors contribute to pain, if any, and defining the patient’s movement goals. Treatment goals are aimed at normalizing a patient’s environmental interactions and engagements, and addressing and mitigating those neuro-musculoskeletal variables contributing to pain.

#### 4.2.2. Integrative Medicine: Active Mind-Body Techniques

Integrative modalities (sometimes referred to as complementary and alternative medicine) that have been described as effective in the management of pediatric pain include hypnosis, yoga, acupuncture, and massage [99,100,101,102,103,104,105,106,107]. Active mind-body techniques, such as guided imagery, hypnosis, biofeedback, yoga, and distraction each and all evoke pain modulation by engaging a number of mechanisms within the analgesic neuraxis. Techniques such as distraction and guided imagery appear to modulate the release of endogenous opioids from the periaqueductal and periventricular grey regions to disinhibit descending inhibitory pathways of the brainstem to suppress pain transmission in the dorsal horn of the spinal cord [108,109,110,111,112]. As well, distraction has been shown to increase activity of the orbitofrontal and perigenual anterior cingulate cortex, as well as periaqueductal grey and the posterior thalamus to modulate pain at the supraspinal level [113,114]. 

In our clinic, integrative and active mind-body techniques are taught to patients, or to the parents, when children are younger than school age, or cognitively impaired. Mind-body techniques focus on intervention strategies that integrate cognitive and emotional processes with physiological functions and experience in order to promote health. We expect all our patients (who are older than five years, and cognitively capable) to learn at least one active integrative medicine technique and incorporate this into their daily routine. Parents are offered training in the same techniques so that they can be aware of what their child is learning, and can reinforce practice as needed. We also expect our patients to learn in our clinic and practice daily at home or school at least one age-appropriate mind-body technique of relaxation and self-regulation. Modalities our patients have reported to be helpful include breathing strategies (e.g., diaphragmatic breathing, square breathing, and “snake”/slow exhale breathing), aromatherapy, biofeedback, progressive muscle relaxation, autogenic training, mindfulness, yoga, and/or self-hypnosis. In addition, we support and offer passive integrative modalities such as acupressure, acupuncture, and/or massage (as supplemental to, but not in place of, self-directed techniques).

#### 4.2.3. Psychological Intervention

Anxiety, depressive, and behavioral disorders are early risk factors of chronic pain (rather than vice versa) [115]. At low levels of anxiety, higher pain is predictive of greater disability; however, highly anxious adolescents tend to function poorly regardless of level of pain [116]. Psychological treatments significantly reduce pain intensity that is reported by children and adolescents with headache, abdominal pain, and musculoskeletal/joint pain [117,118]. Cognitive behavioral therapy (CBT) led to significant improvements in pain coping, catastrophizing, and efficacy that were sustained over time in adolescents with chronic pain [119]. CBT has been shown to increase grey matter in the prefrontal cortex of patients with chronic pain, and this increase in prefrontal cortical grey matter has been associated with reduced pain catastrophizing [120].

In our clinic, psychological intervention is a routine part of our treatment approach, and serves to teach patients general coping skills and integrative medicine strategies, as well as help incorporate these into a daily routine to promote consistent practice, and to promote normalization of a patient’s daily life. Most patients in our clinic experience psychological distress due to disrupted functioning, as secondary to pain, and not as a primary presenting concern. As such, our psychologists’ role is to promote restoration of baseline functioning by teaching appropriate coping strategies and by targeting pain-related fears and catastrophizing that may disrupt normal cognitive and behavioral functions.

#### 4.2.4. Normalizing Life: “The 4 S’s”: Sports, Social, Sleep and School

As previously discussed, we explain to our patients that “first your life gets back to normal, then your pain decreases—unfortunately it’s not the other way around.” We go on to explain “sometimes, pain may even increase, before it gets better.” We explain that the “4 S’s”: sports, socialization, sleep and school need to return to normal parameters before pain resolution could or should be expected.

- Sports: As mentioned in the previous section addressing physical therapy, we place strong emphasis on restoring activity and returning patients to their normal regimen of physical activity, exercise and/or sports.
- Social: When adolescents with chronic pain do not perceive their friends as providing support, they tend to avoid social situations [121]. The social lives of the chronic pain patients in our clinic are commonly disrupted for a variety of reasons, including inability to keep up with peers, disruptions to sports/extra-curricular activities where social contacts occur, experience or fear of being teased because of pain (“you are faking it”) or disability. In our clinic, the family therapist and psychologist work with the patient and his or her family to develop strategies and tactics to regain a balanced social life, provide validation of feeling misunderstood, and continuous medical reassurance that pain is physiologically mediated or “real”.
- Sleep: The majority of children with chronic pain have sleep difficulties, including problems with sleep initiation, sleep maintenance, and/or early morning awakening [122]. These sleep problems tend to be persistent and are associated with negative impact for youths with chronic pain [123]. Treatment of insomnia in youths with chronic pain may lead to improvements in quality of life and reduction in healthcare cost. In our practice, the majority of patients’ parents are successfully coached to assist their children in waking in the morning, having breakfast, attending to personal hygiene, and leaving the house in time to attend the first class at school. We encourage a “no nap” policy, and allow patients to “sleep in” one to two hours later (but no longer) on weekends. We expect that illuminated screens (e.g., television, computer monitors, smart phones, tablets, etc.) will not be used starting one hour before bedtime (or after bedtime), as studies have shown that the blue light emitted by such screens can interfere with melatonin production and/or release [124,125].
- School: Parental catastrophizing and protective responses to their child’s pain predict school attendance rates and overall school performance [126]. Long-term scholastic impairment results in reduced occupational achievement, increased educational costs, and increased risk of developing psychiatric disorders (e.g., anxiety, depression) [127,128,129]. In our clinic, the child’s social worker and psychologist work closely with the patient, parent, and school (if and when permission is granted) to develop a personalized school re-entry plan. Factors considered in developing a re-entry plan include possible learning concerns, stigmatization, teasing and/or bullying by peers, and secondary gain/special attention due to pain and/or disability behavior(s). We have found that with close communication, most schools are supportive of having students take time-limited breaks to practice integrative medicine strategies before returning to the classroom. We typically work with schools for informal accommodations, instead of pursuing an Individualized Education Plan (IEP) or a 504 plan (Section 504 of the USA Rehabilitation Act was developed to guarantee that a child with a disability as identified under the law who is attending an elementary or secondary educational institution will receive accommodations that insure equal capacity to access the learning environment and achieve academic success) to underscore our expectation that the student will return to baseline functioning without accommodation(s).

Parents are asked if they can consistently and successfully get their child/teenager to school regardless of the patient’s report of their inability to go (due to chronic pain). The social worker helps the parent to develop a plan to insure their child’s school attendance, inclusive of conjoining other family members to such tasks, if and as necessary to assist parents.

We usually do not support online school or home schooling for children who have attended a physical facility school prior to their pain.

#### 4.2.5. Parent Coaching

Children with chronic pain often have a negative influence in and upon their family life, can pose a financial burden, both in direct and indirect costs incurred from healthcare utilization and lost wages due to parents taking time off work to care for the child, and the child in pain can exert considerable emotional toll on family members. Families of children with chronic pain generally have poorer family functioning, and pain-related disability is more consistently related to family functioning than pain intensity [130]. Studies have shown that mothers of children with chronic abdominal pain show pain bias when interpreting ambiguous emotional expressions, and such bias might possibly contribute to parenting behaviors that maintain or enhance the child’s pain [131]. Children of parents with chronic pain have been shown to display poorer outcomes in health, as well as psychological and familial functioning [132], and show increased risk of developing anxiety and depression as teenagers [133].

Parents in our clinic tend to be hypervigilant and pay increased attention to their children’s pain reports/behaviors, given that they commonly are asked to report on pain specifics and details during routine medical consults. As such, parents may accidentally reinforce their child’s pain behaviors. It is our clinical experience that parents are often defensive about their role in (maintaining) their child’s pain. Normalizing parental protectiveness is a helpful first step toward shifting parents’ focus to attend to healthy, adaptive behaviors, and decreasing their attention to their child’s pain behaviors. We often ask parents to focus on their child’s functioning and ask about specific daily activities, instead of attending to pain-related disruptions of their child’s functioning. Depending on the child’s age and developmental level, we may encourage parents to actively model mind-body and integrative medicine strategies to help their child engage in active and appropriate coping.

Parents’ experiences of/with chronic pain and disability can influence their children’s pain beliefs and behaviors. Parents’ pain experiences are openly addressed and discussed with both the child and parent(s). The social worker discusses how the patient’s pain is different from the parent’s disability and pain, and the expectation that the child can become pain-free. The message we seek to communicate is that there is no reason to believe that they will be disabled like their parent.

Parents are often taught some of the same skills that are taught to their child/teenager by the psychologist. The social worker does this in order to teach the parents how to calm themselves when their child is having a pain flare. The parent is encouraged to use such skills to avoid incurring emotional escalations when dealing with their child. We believe that the parent is in an ideal position to emotionally anchor their child and support the use of calming skills to reduce pain levels. It is also important to teach parents to gently remind their child that normalization of daily functioning must come first before pain starts to decrease, and that progress is measured initially by the patient’s return to a normalized daily life for sports, social life, sleep and school. Indeed it is easy for both the patient and parent to lose sight of this when the child continues to experience and present with pain. While active engagement of the parent(s) is advocated when working with younger children, a different approach is used when the patient is a teenager. Teenagers are taught and encouraged to acquire, develop and employ such skills more independently, and to foster greater capabilities for independence in activities of daily living. It has been our experience that parents are usually motivated to change any maladaptive or deleterious behavior. Clearly, they are very distressed by both seeing their child in pain and not knowing how to help them. The skills provided by our approach give the parent focus and a set of tasks, which can reduce their sense of helplessness and decrease their catastrophizing, while concomitantly helping their child. 

## 5. Medications

In our pediatric pain practice we commonly find medications as a sole therapy for primary pain disorders to be ineffective, especially if they are not accompanied by the aforementioned modalities of our rehabilitative pain program. Still, it is important to consider the validity and value of pharmacological interventions as part of an integrative approach to pediatric pain care. In this regards some pharmacological considerations are included below.

### 5.1. Basic Analgesics: Acetaminophen and Ibuprofen

For mild, acute pain (i.e., associated with tissue injury), acetaminophen and ibuprofen are the agents of choice [80]. No other NSAID has been sufficiently studied for efficacy and safety in the pediatric population so as to be recommended as an alternative to ibuprofen. Although there is evidence for the superior analgesic properties of ibuprofen versus acetaminophen, it is considered to be of limited value because the studies were mostly performed in acute pain settings and lack long-term safety data. Both acetaminophen and ibuprofen have potential toxicities. There are concerns about renal and gastrointestinal toxicity, and bleeding with ibuprofen and other NSAIDs, and risks of hepatotoxicity and acute overdose are associated with acetaminophen [80]. Celecoxib (a cyclooxygenase-2 (COX-2) enzyme inhibitor) might be considered, if classical NSAIDs are contraindicated (e.g., owing to bleeding risks, or gastrointestinal side effects). Celecoxib does not display less renal toxicity as compared to classic NSAIDs. Safety and efficacy have been established only in children two years of age or older with juvenile rheumatoid arthritis, and for a maximum of six months of treatment.

With the exception of ibuprofen’s proven efficacy in reducing pain at the onset of a migraine episode [50,51,52], basic analgesics generally are not used to treat primary pain disorders in the absence of new tissue injury. As discussed earlier, daily use of analgesics in headache patients incurs significant risk of leading to medication overuse headaches (“rebound headaches”).

#### Fast-Acting NSAID: Ibuprofen-Sodium

Data have shown that when compared to ibuprofen, ibuprofen-sodium produced significantly greater analgesia over 6 h, and required fewer re-medications than standard formulations [134]. In addition, 200 mg fast-acting ibuprofen (Numbers-needed-to-treat (NNT) 2.1; 95% confidence interval (CI) 1.9–2.4) was as effective as 400 mg standard ibuprofen (NNT 2.4; 95% CI 2.2–2.5), and produced a faster onset of analgesia. Meta-analysis showed that NSAID-salts display more rapid absorption, faster initial pain reduction, good overall analgesia in more patients at the same dose, and probably evoke longer-lasting analgesia, without reports of adverse events [135]. For example, in our practice, we suggest fast-acting ibuprofen plus acetaminophen as a first step, followed by a triptan, if ineffective, be utilized within 10 min of onset of a migraine attack. In the USA, ibuprofen-sodium is available over the counter for about $8–$10 for 80 tablets (containing 266 mg of ibuprofen sodium, which is the equivalent to 200 mg of standard ibuprofen).

### 5.2. Opioids

The Center for Disease Control and Prevention’s recently published 2016 Guidelines for Prescribing Opioids for Chronic Pain [136] do not apply to children and teenagers [137] and encompass only “patients aged ≥18 years with chronic pain outside of palliative and end-of-life care.” The guidelines state further that the “recommendations do not address the use of opioid pain medication in children or adolescents aged <18 years”.

Opioids administered for primary pain disorders have low long-term efficacy, a poor safety profile, and commonly a worse clinical outcome [133,134,135,136,137,138,139]. Thus, opioids should not be administered to pediatric patients with primary pain disorders [43], i.e., chronic pain defined that extends beyond the expected time of healing and hence lacks the acute warning function of physiological nociception. Opioids may be more likely to cause more harm than benefit in the treatment of primary pain disorders, which include conditions such as tension-type/migraines headaches, chronic musculoskeletal pain, “chronic sickle cell pain” (pain that extends beyond the expected time of acute vaso-occlusive crisis) and centrally mediated abdominal pain syndrome. In our practice, we do not prescribe opioids for primary pain disorders in children or teenagers, as we consider them to be contraindicated. On the other hand, in persistent pain conditions (i.e., long-lasting and/or repetitive nociceptive pain caused by tissue injury, such as in children with junctional epidermolysis bullosa, osteogenesis imperfecta, or advanced metastasized bone tumors (e.g., Ewing sarcoma)) opioids are important and effective for long-term analgesic management.

### 5.3. Adjuvant Analgesics

Adjuvant analgesics (e.g., low-dose tricyclic antidepressants, gabapentinoids, α-agonists, melatonin, etc.) may serve as valuable adjuncts. Although commonly used for primary pain disorders, there is little evidence to support their use against pediatric pain. Most pediatric data for this heterogeneous class of medications is derived from neuropathic and acute pain conditions [138,139,140,141,142,143,144]. The most commonly prescribed adjuvant analgesics in our pain clinic are melatonin, lidocaine 5% patch, gabapentin, and low-dose amitriptyline (See Table 5).

For migraines, only ibuprofen and triptans are supported by sufficient evidence to engender use in pediatrics [50,51,52]. Medication to treat migraines in adolescents have a strong placebo effect, with pain relief at 2 h in the placebo arms of seven randomized controlled trials ranging from 53% to 57.5%. A meta-analysis of 21 randomized controlled trials (RCTs) of pediatric headache management [145] revealed that placebo decreased headache frequency from 5.6 to 2.9 headaches/month, and also most commonly used drugs had little to no evidence to support their use in children and adolescents. Drugs shown to be ineffective included clonidine, flunarizine, pizotifen, propranolol, valproate, and fluoxetine [145].

### 5.4. Selective Serotonin Re-Uptake Inhibitors (SSRI)/Serotonin and Norepinephrine Reuptake Inhibitors (SNRI)

There is little evidence to support the use of SSRIs or SNRIs in children/teenagers with primary pain disorders. An over-quoted, uncontrolled open-label case series for example has claimed that 21 of 25 children with recurrent abdominal pain improved following SSRI treatment [146]. However, placebo effects were large with pediatric antidepressant trials in other settings, regression to the mean must be expected, and there might be inherent bias at play. Efficacy studies for duloxetine in pediatric pain patients are lacking. In our clinic practice, we add an SSRI (such as citalopram, sertraline, escitalopram) in addition to (not in substitution for) individual psychotherapy to the treatment regimen of some patients who present with an underlying mental health diagnosis of anxiety and/or depression. 

### 5.5. Laxatives

Constipation is the most common diagnosis in children presenting with abdominal pain in the emergency room [147], and in our practice, we see a surprisingly large number of children with long-standing abdominal pain, who have constipation and/or overflow diarrhea despite having been seen by many clinicians and reports that they have tried many laxatives. In our practice, we nearly always employ a scheduled (not “as needed” or pro re nate “prn”)) regimen of (1) “Mush” (stool softener such as lactulose or polyethylenglycol 3350); (2) “Push” (stimulant such as senna) and (3) “Uncorking” (glycerin suppository for children <4 years, and bisodacyl suppository for older children) if no stool has been evacuated in the previous 24 h. This regimen has proven to be highly effective in resolving constipation, thereby decreasing or resolving abdominal pain [148].

### 5.6. Multimodal (”Opioid-Sparing”) Analgesia

Multimodal analgesia is an approach used to prevent and treat pain in children: multiple agents, interventions, rehabilitation, and psychological and integrative therapies often act synergistically to elicit more effective pediatric pain control with fewer side effects than a single analgesic or modality [149]. Multimodal analgesic therapy (versus opioids alone) has been shown to reduce the length of hospitalization in patients undergoing surgery [150].

Evidence-based, safe multimodal (i.e., opioid-sparing) analgesia may include one, several or all of the following approaches: pharmacology (e.g., simple analgesia and/or opioids and/or adjuvant analgesia), anesthetic interventions (e.g., neuroaxial analgesia, nerve blocks), rehabilitation (e.g., physical therapy, occupational therapy, sleep hygiene), psychological counseling (e.g., cognitive behavioral therapy), and age-appropriate positioning and integrative (non-pharmacological) therapies, such as breathing techniques, self-hypnosis, and distraction.

## 6. From Mechanism and Classifications to Practice: Obligations for Care

Taken together, the use of these approaches has been shown to be highly effective in treating primary pain disorder in our clinical experience. Our approach is based and built upon a growing body of information that is both revising extant concepts of pain and pain modulation, and exploring new approaches to the assessment and treatment of pain. To be sure, new insights and perspectives about mechanisms and categorizations of pain in general, and pediatric pain in particular, are indubitably important to provide better understanding of pathology and more precise nosology [151,152]. Large-scale initiatives, such as the ongoing, federally directed Brain Research through Advancing Innovative Neurotechnologies (BRAIN) [153], and precision medicine initiatives, are developing more finely grained approaches to research, which seek to better define the neurobiology and psychosocial aspects of pediatric pain. Such knowledge must be appreciated and employed for its practical utility toward improving diagnosis and treatment of the pain patient [154].

As we have stated previously, and reaffirm here: “ … we … study pain so that we may unravel its mysteries and develop more and better ways of relieving pain...to restore and sustain … living a life unencumbered by suffering” [155]. Understanding the bio-psychosocial dimensions of pain—as symptom, pathology and manifest illness—compels providing a more comprehensive and integrative approach to both assessment and treatment [156,157]. Such an approach entails the coordinated use of multiple disciplines, and engages both “high tech” and “low tech” means [158]. This enables both a more thorough evaluation of each patient’s needs (in physiological, psychological and social dimensions), and more personalized, evidence-based intervention(s) to lessen or eradicate pain, and its manifest effects.

## 7. From Concepts to Practice: Our Experience at a Glimpse

Our interdisciplinary, rehabilitative pediatric pain clinic at Children’s Minnesota has been seeing pediatric patients since 2006. Based on a 2007–2009 three-year prospective study (*n* = 145) and two chart reviews (2010 and 2014, *n* = 135) by Desai et al. [159,160] the majority (74%) of our new patients are female with an average age of 14 years (range ten-day-old infant to 17 years). The most common presenting conditions are listed in Table 6.

On average, our patients’ pain began 2.4 years prior to intake and 57% of our patients had an immediate family member with chronic pain. On a pain scale 0–10, with 10 being the worst, our patients’ average daily pain was rated 5.6/10 (worst: 8.8/10, least bad 3/10). A total of 75% of our pain patients also received a mental health diagnosis; 67% presented with anxiety (including general anxiety disorder, panic episodes, anxiety disorder not otherwise specified (NOS)), and 30% presented with depression (including major depression, and depressive disorder NOS) [160]. 

In reviewing our data from 2007 to 2009, 2010, and 2014, it appears that between 83% and 92% of new patients chose to follow-up with the rehabilitative treatment recommendations provided by our clinic after the initial intake. Of those, the majority (67%–79%) graduated successfully, (i.e., are pain-free or pain-free most of the time). Of the remaining, up to 26% were referred to additional and/or separate services (e.g., inpatient or outpatient psychiatry, eating disorder program, physical therapy/mental health provider closer to home) and of our most recent data review, 13% were still in treatment 6–12 months later [159]. The patients who successfully graduated had seen the physical therapist eight (mean; range 1–25) times, psychologist ten times (range 1–34), parents the family therapist/social four times (range 1–16), and the physician/nurse practitioner 2.5 times (range 1–7).

As previously noted, in our clinic at the first visit only 16% of children reported that they believed they could ever become pain-free. However, at the exit interview following the first visit, 92% of children asserted that they believed being pain-free was possible. The rehabilitative interdisciplinary treatment approach does not only seem to be effective for patients with primary pain disorders and CRPS, but also, in addition to other multimodal analgesia strategies, for the treatment of chronic-on-acute pain or chronic-on-neuropathic pain patients (see Section 3.6), indicating the significant overlap these conditions may have.

The work of our interdisciplinary team can be seen in a short movie, “Little Stars—Treating Chronic Pain in Children [161].”

## 8. Conclusions

Primary pain disorder (what was formerly known as “functional pain syndrome”) is a common, under-diagnosed and under-treated condition in pediatric patients. Primary pain disorder occurs as a mechanistic vulnerability to multi-focal pain that can occur and be expressed at a number of bodily sites. Common symptoms of this underlying pain vulnerability include primary headache, centrally mediated abdominal pain syndrome, and chronic/recurrent musculoskeletal pain. A significant number of children with repeated acute nociceptive pain episodes develop chronic pain in addition to or as a result of their condition. Untreated chronic pediatric pain can increase the risk of pain, as well as physical and psychiatric disorders in adulthood. These findings reinforce the importance, if not obligation, to effectively address and treat pain in children and adolescents.

In our interdisciplinary pediatric pain management practice, we have shown that an integrative, multidisciplinary approach combining rehabilitation, complementary therapies (e.g., distraction, hypnosis, etc.), psychological counseling, and normalization of physical/sports activity, sleep, socialization, and school attendance—together with the prudent use of pharmacological agents—effectively mitigates and/or commonly resolves our patients’ pain. Of note, however, is that opioids are not indicated for primary pain disorders, and other medications do not usually represent first-line therapy.

Further Resources for Clinicians and Patients

(1) Pediatric Pain Clinics in USA and Canada (American Pain Society): http://americanpainsociety.org/uploads/get-involved/PainClinicList_12_2015.pdf [162].
(2) Short Movie: Meet the Interdisciplinary Chronic Pain Clinic Team at Children’s Minnesota: LittleStars TV https://www.youtube.com/watch?t=13&v=Bb1fHxfjdWI [161].
(3) Pain Bytes (Australia) [163].
(4) Persistent (Chronic) Pain 5-min video [164].
(5) Kiran Stordalen and Horst Rechelbacher Pediatric Pain, Palliative and Integrative Medicine Clinic Tour [165].
(6) Elliot Krane (TED-Talk) The mystery of chronic pain [166].
(7) The Department of Pain Medicine, Palliative Care, and Integrative Medicine, Children’s Hospitals and Clinics of Minnesota [167].

## Figures and Tables

**Table 1:** Headache warning signals requiring further workup, including neuroimaging.

Focal or abnormal neurological signs, ataxia Papilledema (including rule out pseudotumor cerebri) Age < 3 years “Worst headache of my life” Progressive worsening headaches Ventriculoperitoneal-shunt Neurocutaneous syndrome Immunocompromised → Cerebrospinal fluid? (check with Infectious disease, oncology or transplant clinician)

Rule out: carbon monoxide toxicity; Obstructive sleep apnea.

**Table 2:** Abdominal pain warning signals requiring further workup.

Persistent right upper or right lower quadrant pain Pain that wakes child from sleep Dysphagia Arthritis Persistent vomiting Perirectal disease Gastrointestinal blood loss Involuntary weight loss Nocturnal diarrhea Deceleration of linear growth Unexplained fever

**Table 3:** Musculoskeletal/joint pain warning signals requiring further workup.

Arthralgia: Rubor, calor, edema Pain/stiffness in the morning Abnormal radiographic findings Pain at rest, relieved by activity Pain at night: Worsened by massage, analgesics ineffective Bony tenderness Poor growth Weight loss Abnormal blood results: Including complete blood count (CBC), C-reactive protein (CRP), erythrocyte sedimentation rate (ESR)

**Table 4:** Reasons to refer a pediatric chronic pain patient to physical therapy.

Goals of returning to sport or activities Not participating in gym class Signs of weakness, poor balance, poor endurance, abnormal movement patterns, or poor posture, etc. Diagnoses associated with abnormal movement patterns or weakness: e.g., Ehlers–Danlos syndrome, complex regional pain syndrome (CRPS), centrally mediated abdominal pain syndrome (CAPS), chronic musculoskeletal pain, chronic headaches, etc.

**Table 5:** Adjuvant analgesics used in pediatric pain management (Pain Medicine and Palliative Care, Children’s Hospitals and Clinics of Minnesota) [79].

Class	Medication	Dose	Route of Administration	Comments/Side Effects (See Text for Further Details)
**Tricyclic Antidepressants (TCA)**	**Amitriptyline**	Starting dose 0.1 mg/kg QHS, usually slowly titrated up to 0.5 mg/kg (max. 20-25 mg)	PO	Tertiary amine TCA; stronger anticholinergic side effects (including sedation) than nortriptyline
	**Nortriptyline**	Starting dose 0.1 mg/kg QHS, usually titrated up to 0.5 mg/kg (max. 20-25 mg)	PO	Secondary amine TCA; anticholinergic side effects
**Gabapentenoids**	**Gabapentin**	Starting dose 2 mg/kg QHS, usually slowly titrated up to initial target dose of 6 mg/kg/dose TID (max. 300 mg/dose TID). Max. dose escalation to 24 mg/kg/dose TID (max. 1200 mg/dose TID)	PO	Slow dose increase required; side effects: ataxia, nystagmus, myalgia, hallucination, dizziness, somnolence, aggressive behaviors, hyperactivity, thought disorder, peripheral edema
	**Pregabaline**	Starting dose 0.3 mg/kg QHS, usually slowly titrated up to initial target dose of 1.5 mg/kg/dose BID (max. 75 mg/dose BID). Max. dose escalation to 6 mg/kg/dose BID (max. 300 mg/dose BID)	PO	Switch from gabapentin, if distressing side effects or inadequate analgesia. Side effects: ataxia, nystagmus, myalgia, hallucination, dizziness, somnolence, aggressive behaviors, hyperactivity, thought disorder, peripheral edema; Associated with weight gain
**Sodium Channel Blocker/Local anesthetic**	**Lidocaine 5%**	Max. of 4 patches (in patients > 50 kg) 12 h on/12 h off	Transdermal patch	Not for severe hepatic dysfunction
**Alpha-Agonist**	**Clonidine**	1–3 mcg/kg QHS to Q6h	PO/transdermal	
		
	**Dexmedetomidine**	Infusion: 0.3 mcg/kg/h; titrate to max. 2 mcg/kg/h	IV	
**Hormone**	**Melatonin**	0.06–0.2 mg/kg (max. 3–10 mg) QHS	PO	Sleep induction, use extended-release, if interrupted sleep, possible analgesic effect

QHS: every night at bedtime; PO: per os, oral administration; IV: intravenous administration; BID: bis in die, twice a day; TID: ter in die, three times a day; Q6h: every 6h.

**Table 6:** Presenting or accompanying pain conditions: Initial intake at interdisciplinary pediatric pain clinic at Children’s Minnesota.

74% Chronic or recurrent musculoskeletal pain 61% Primary headaches (tension-type/migraines) 38% CAPS 11% CRPS type I 26% Additional or accompanying underlying conditions, including Avascular necrosis Caffe’s disease Cerebral palsy/spasticity Chiari-I-malformation with ventricular-peritoneal (VP) shunt Chronic postsurgical pain Conversion disorder CRPS type 2 Erythromelalgi Inflammatory bowel disease (Crohn’s disease, ulcerative colitis) Irritable bowel syndrome Juvenile rheumatoid/idiopathic arthritis (JRA/JIA) Malignancy Muscular dystrophy Penilodynia Phantom limb pain Progressive neurodegenerative/metabolic conditions incl. mitochondriopathies Sickle cell disease Vulvodynia

## References

[1] Goodman J.E., McGrath P.J. (1991). The epidemiology of pain in children and adolescents: A review. Pain.

[2] King S., Chambers C.T., Huguet A., MacNevin R.C., McGrath P.J., Parker L., MacDonald A.J. (2011). The epidemiology of chronic pain in children and adolescents revisited: A systematic review. Pain.

[3] Stanford E.A., Chambers C.T., Biesanz J.C., Chen E. (2008). The frequency, trajectories and predictors of adolescent recurrent pain: A population-based approach. Pain.

[4] Friedrichsdorf S.J., Postier A., Eull D., Weidner C., Foster L., Gilbert M., Campbell F. (2015). Pain outcomes in a US children’s hospital: A prospective cross-sectional survey. Hosp. Pediatr..

[5] Taylor E.M., Boyer K., Campbell F.A. (2008). Pain in hospitalized children: A prospective cross-sectional survey of pain prevalence, intensity, assessment and management in a Canadian pediatric teaching hospital. Pain Res. Manag..

[6] Zhu L.M., Stinson J., Palozzi L., Weingarten K., Hogan M.E., Duong S., Carbajal R., Campbell F.A., Taddio A. (2012). Improvements in pain outcomes in a Canadian pediatric teaching hospital following implementation of a multifaceted knowledge translation initiative. Pain Res. Manag. J. Can. Pain Soc..

[7] Stevens B.J., Harrison D., Rashotte J., Yamada J., Abbott L.K., Coburn G., Stinson J., Le May S. (2012). Pain assessment and intensity in hospitalized children in canada. J. Pain.

[8] Huguet A., Miro J. (2008). The severity of chronic pediatric pain: An epidemiological study. J. Pain.

[9] Hechler T., Dobe M., Zernikow B. (2010). Commentary: A worldwide call for multimodal inpatient treatment for children and adolescents suffering from chronic pain and pain-related disability. J. Pediatr. Psychol..

[10] Groenewald C.B., Essner B.S., Wright D., Fesinmeyer M.D., Palermo T.M. (2014). The economic costs of chronic pain among a cohort of treatment-seeking adolescents in the United States. J. Pain.

[11] Hyams J.S., di Lorenzo C., Saps M., Shulman R.J., Staiano A., van Tilburg M. (2016). Functional disorders: Children and adolescents. Gastroenterology.

[12] Turk D., Okifuji A., Bonica J., Loeser J., Chapman C., Turk D., Butler S. (2001). Pain terms and taxonomies of pain. Bonica’s Management of Pain.

[13] Treede R.D., Rief W., Barke A., Aziz Q., Bennett M.I., Benoliel R., Cohen M., Evers S., Finnerup N.B., First M.B. (2015). A classification of chronic pain for ICD-11. Pain.

[14] Force A.P.S.P.C.P.T. Assessment and Management of Children with Chronic Pain. A Position Statement from the American Pain Society. http://americanpainsociety.org/uploads/get-involved/pediatric-chronic-pain-statement.pdf.

[15] Woolf C.J. (2011). Central sensitization: Implications for the diagnosis and treatment of pain. Pain.

[16] Special Issue “Chronic and Recurrent Pain”. http://www.mdpi.com/journal/children/special_issues/chronic_pain.

[17] Hassett A.L., Hilliard P.E., Goesling J., Clauw D.J., Harte S.E., Brummett C.M. (2013). Reports of chronic pain in childhood and adolescence among patients at a tertiary care pain clinic. J. Pain.

[18] Johannes C.B., Le T.K., Zhou X., Johnston J.A., Dworkin R.H. (2010). The prevalence of chronic pain in United States adults: Results of an internet-based survey. J. Pain.

[19] Hotopf M., Mayou R., Wadsworth M., Wessely S. (1999). Psychosocial and developmental antecedents of chest pain in young adults. Psychosom. Med..

[20] Hotopf M., Carr S., Mayou R., Wadsworth M., Wessely S. (1998). Why do children have chronic abdominal pain, and what happens to them when they grow up? Population based cohort study. BMJ.

[21] Jones G.T., Silman A.J., Power C., Macfarlane G.J. (2007). Are common symptoms in childhood associated with chronic widespread body pain in adulthood? Results from the 1958 British birth cohort study. Arthritis Rheum..

[22] Power C., Elliott J. (2006). Cohort profile: 1958 British birth cohort (National Child Development Study). Int. J. Epidemiol..

[23] Mulvaney S., Lambert E.W., Garber J., Walker L.S. (2006). Trajectories of symptoms and impairment for pediatric patients with functional abdominal pain: A 5-year longitudinal study. J. Am. Acad. Child Adolesc. Psychiatry.

[24] Dunn K.M., Jordan K.P., Mancl L., Drangsholt M.T., Le Resche L. (2011). Trajectories of pain in adolescents: A prospective cohort study. Pain.

[25] Horst S., Shelby G., Anderson J., Acra S., Polk D.B., Saville B.R., Garber J., Walker L.S. (2014). Predicting persistence of functional abdominal pain from childhood into young adulthood. Clin. Gastroenterol. Hepatol..

[26] Shelby G.D., Shirkey K.C., Sherman A.L., Beck J.E., Haman K., Shears A.R., Horst S.N., Smith C.A., Garber J., Walker L.S. (2013). Functional abdominal pain in childhood and long-term vulnerability to anxiety disorders. Pediatrics.

[27] Noel M., Groenewald C.B., Beals-Erickson S.E., Gebert J.T., Palermo T.M. (2016). Chronic pain in adolescence and internalizing mental health disorders: A nationally representative study. Pain.

[28] Van Tilburg M.A., Spence N.J., Whitehead W.E., Bangdiwala S., Goldston D.B. (2011). Chronic pain in adolescents is associated with suicidal thoughts and behaviors. J. Pain.

[29] Fisher E., Palermo T.M. (2016). Goal pursuit in youth with chronic pain. Children.

[30] Cunningham N.R., Lynch-Jordan A., Barnett K., Peugh J., Sil S., Goldschneider K., Kashikar-Zuck S. (2014). Child pain catastrophizing mediates the relation between parent responses to pain and disability in youth with functional abdominal pain. J. Pediatr. Gastroenterol. Nutr..

[31] Lynch-Jordan A.M., Kashikar-Zuck S., Szabova A., Goldschneider K.R. (2013). The interplay of parent and adolescent catastrophizing and its impact on adolescents’ pain, functioning, and pain behavior. Clin. J. Pain.

[32] Williams S.E., Blount R.L., Walker L.S. (2011). Children’s pain threat appraisal and catastrophizing moderate the impact of parent verbal behavior on children’s symptom complaints. J. Pediatr. Psychol..

[33] Campbell C.M., Witmer K., Simango M., Carteret A., Loggia M.L., Campbell J.N., Haythornthwaite J.A., Edwards R.R. (2010). Catastrophizing delays the analgesic effect of distraction. Pain.

[34] Birnie K.A., Chambers C.T., Chorney J., Fernandez C.V., McGrath P.J. (2016). Dyadic analysis of child and parent trait and state pain catastrophizing in the process of Children’s Pain Communication. Pain.

[35] Noel M., Rabbitts J.A., Tai G.G., Palermo T.M. (2015). Remembering pain after surgery: A longitudinal examination of the role of pain catastrophizing in children’s and parents’ recall. Pain.

[36] Simons L.E., Kaczynski K.J., Conroy C., Logan D.E. (2012). Fear of pain in the context of intensive pain rehabilitation among children and adolescents with neuropathic pain: Associations with treatment response. J. Pain.

[37] Zale E.L., Lange K.L., Fields S.A., Ditre J.W. (2013). The relation between pain-related fear and disability: A meta-analysis. J. Pain.

[38] Von Baeyer C.L., Champion G.D. (2011). Commentary: Multiple pains as functional pain syndromes. J. Pediatr. Psychol..

[39] Kindler L.L., Bennett R.M., Jones K.D. (2011). Central sensitivity syndromes: Mounting pathophysiologic evidence to link fibromyalgia with other common chronic pain disorders. Pain Manag. Nurs..

[40] Williams F.M., Spector T.D., MacGregor A.J. (2010). Pain reporting at different body sites is explained by a single underlying genetic factor. Rheumatology.

[41] Mayer E.A., Bushnell M.C. (2009). Functional Pain Syndromes: Presentation and Pathophysiology.

[42] Burri A., Ogata S., Vehof J., Williams F. (2015). Chronic widespread pain: Clinical comorbidities and psychological correlates. Pain.

[43] Schechter N.L. (2014). Functional pain: Time for a new name. JAMA Pediatr..

[44] McAbee G.N., Morse A.M., Assadi M. (2016). Pediatric aspects of headache classification in the international classification of headache disorders-3 (ichd-3 beta version). Curr. Pain Headache Rep..

[45] Headache Classification Committee of the International Headache Society (2013). The International Classification of Headache Disorders, 3rd Edition (Beta Version). Cephalalgia Int. J. Headache.

[46] Viswanathan V., Bridges S.J., Whitehouse W., Newton R.W. (1998). Childhood headaches: Discrete entities or continuum?. Dev. Med. Child Neurol..

[47] Zebenholzer K., Wober C., Kienbacher C., Wober-Bingol C. (2000). Migrainous disorder and headache of the tension-type not fulfilling the criteria: A follow-up study in children and adolescents. Cephalalgia Int. J. Headache.

[48] Turner D.P., Smitherman T.A., Black A.K., Penzien D.B., Porter J.A., Lofland K.R., Houle T.T. (2015). Are migraine and tension-type headache diagnostic types or points on a severity continuum? An exploration of the latent taxometric structure of headache. Pain.

[49] Andersen S., Petersen M.W., Svendsen A.S., Gazerani P. (2015). Pressure pain thresholds assessed over temporalis, masseter, and frontalis muscles in healthy individuals, patients with tension-type headache, and those with migraine—A systematic review. Pain.

[50] Sun H., Bastings E., Temeck J., Smith P.B., Men A., Tandon V., Murphy D., Rodriguez W. (2013). Migraine therapeutics in adolescents: A systematic analysis and historic perspectives of triptan trials in adolescents. JAMA Pediatr..

[51] Silver S., Gano D., Gerretsen P. (2008). Acute treatment of paediatric migraine: A meta-analysis of efficacy. J. Paediatr. Child Health.

[52] Damen L., Bruijn J.K., Verhagen A.P., Berger M.Y., Passchier J., Koes B.W. (2005). Symptomatic treatment of migraine in children: A systematic review of medication trials. Pediatrics.

[53] Sun-Edelstein C., Bigal M.E., Rapoport A.M. (2009). Chronic migraine and medication overuse headache: Clarifying the current international headache society classification criteria. Cephalalgia Int. J. Headache.

[54] Bigal M.E., Tepper S.J., Sheftell F.D., Rapoport A.M., Lipton R.B. (2004). Chronic daily headache: Correlation between the 2004 and the 1988 international headache society diagnostic criteria. Headache.

[55] Limmroth V., Katsarava Z., Fritsche G., Przywara S., Diener H.C. (2002). Features of medication overuse headache following overuse of different acute headache drugs. Neurology.

[56] MacGregor E.A., Steiner T.J., Davies P.T.G. Guidelines for All Healthcare Professionals in the Diagnosis and Management of Migraine, Tension-Type, Cluster and Medication-Overuse Headache.

[57] Hering-Hanit R., Gadoth N., Cohen A., Horev Z. (2001). Successful withdrawal from analgesic abuse in a group of youngsters with chronic daily headache. J. Child Neurol..

[58] Keefer L., Drossman D.A., Guthrie E., Simren M., Tillisch K., Olden K., Whorwell P.J. (2016). Centrally mediated disorders of gastrointestinal pain. Gastroenterology.

[59] Williams A.E., Heitkemper M., Self M.M., Czyzewski D.I., Shulman R.J. (2013). Endogenous inhibition of somatic pain is impaired in girls with irritable bowel syndrome compared with healthy girls. J. Pain.

[60] Stabell N., Stubhaug A., Flaegstad T., Mayer E., Naliboff B.D., Nielsen C.S. (2014). Widespread hyperalgesia in adolescents with symptoms of irritable bowel syndrome: Results from a large population-based study. J. Pain.

[61] Walker L.S., Dengler-Crish C.M., Rippel S., Bruehl S. (2010). Functional abdominal pain in childhood and adolescence increases risk for chronic pain in adulthood. Pain.

[62] Dengler-Crish C.M., Horst S.N., Walker L.S. (2011). Somatic complaints in childhood functional abdominal pain are associated with functional gastrointestinal disorders in adolescence and adulthood. J. Pediatr. Gastroenterol. Nutr..

[63] Dengler-Crish C.M., Bruehl S., Walker L.S. (2011). Increased wind-up to heat pain in women with a childhood history of functional abdominal pain. Pain.

[64] Petersen S., Bergstrom E., Brulin C. (2003). High prevalence of tiredness and pain in young schoolchildren. Scand. J. Public Health.

[65] Watson K.D., Papageorgiou A.C., Jones G.T., Taylor S., Symmons D.P., Silman A.J., Macfarlane G.J. (2002). Low back pain in schoolchildren: Occurrence and characteristics. Pain.

[66] Kristjansdottir G. (1996). Prevalence of self-reported back pain in school children: A study of sociodemographic differences. Eur. J. Pediatr..

[67] Smedbraten B.K., Natvig B., Rutle O., Bruusgaard D. (1998). Self-reported bodily pain in schoolchildren. Scand. J. Rheumatol..

[68] Brun Sundblad G.M., Saartok T., Engstrom L.M. (2007). Prevalence and co-occurrence of self-rated pain and perceived health in school-children: Age and gender differences. Eur. J. Pain.

[69] Vahasarja V. (1995). Prevalence of chronic knee pain in children and adolescents in Northern Finland. Acta Paediatr..

[70] Mikkelsson M., Salminen J.J., Kautiainen H. (1997). Non-specific musculoskeletal pain in preadolescents. Prevalence and 1-year persistence. Pain.

[71] Centers for Disease Control and Prevention Post-Treatment Lyme Disease Syndrome. http://www.cdc.gov/lyme/postlds/.

[72] U.S. National Library of Medicine. Conversion Disorder. https://medlineplus.gov/ency/article/000954.htm.

[73] Cottencin O. (2014). Conversion disorders: Psychiatric and psychotherapeutic aspects. Neurophysiol. Clin..

[74] Lu Q., Krull K.R., Leisenring W., Owen J.E., Kawashima T., Tsao J.C., Zebrack B., Mertens A., Armstrong G.T., Stovall M. (2011). Pain in long-term adult survivors of childhood cancers and their siblings: A report from the childhood cancer survivor study. Pain.

[75] Fortier M.A., Chou J., Maurer E.L., Kain Z.N. (2011). Acute to chronic postoperative pain in children: Preliminary findings. J. Pediatr. Surg..

[76] Page M.G., Stinson J., Campbell F., Isaac L., Katz J. (2013). Identification of pain-related psychological risk factors for the development and maintenance of pediatric chronic postsurgical pain. J. Pain Res..

[77] Sieberg C.B., Simons L.E., Edelstein M.R., DeAngelis M.R., Pielech M., Sethna N., Hresko M.T. (2013). Pain prevalence and trajectories following pediatric spinal fusion surgery. J. Pain.

[78] Rabbitts J.A., Zhou C., Groenewald C.B., Durkin L., Palermo T.M. (2015). Trajectories of postsurgical pain in children: Risk factors and impact of late pain recovery on long-term health outcomes after major surgery. Pain.

[79] Friedrichsdorf S.J. (2016). Prevention and treatment of pain in hospitalized infants, children, and teenagers: From myths and morphine to multimodal analgesia. Proceedings of the Pain 2016: Refresher Courses, 16th World Congress on Pain.

[80] World Health Organization WHO-Principles of Acute Pain Management for Children. http://apps.who.int/iris/bitstream/10665/44540/1/9789241548120_Guidelines.pdf.

[81] Guzman J., Esmail R., Karjalainen K., Malmivaara A., Irvin E., Bombardier C. (2002). Multidisciplinary bio-psycho-social rehabilitation for chronic low back pain. Cochrane Database Syst. Rev..

[82] Logan D.E., Carpino E.A., Chiang G., Condon M., Firn E., Gaughan V.J., Hogan M., Leslie D.S., Olson K., Sager S. (2012). A day-hospital approach to treatment of pediatric complex regional pain syndrome: Initial functional outcomes. Clin. J. Pain.

[83] Hechler T., Ruhe A.K., Schmidt P., Hirsch J., Wager J., Dobe M., Krummenauer F., Zernikow B. (2014). Inpatient-based intensive interdisciplinary pain treatment for highly impaired children with severe chronic pain: Randomized controlled trial of efficacy and economic effects. Pain.

[84] Eccleston C., Malleson P.N., Clinch J., Connell H., Sourbut C. (2003). Chronic pain in adolescents: Evaluation of a programme of interdisciplinary cognitive behaviour therapy. Arch. Dis. Child.

[85] Maynard C.S., Amari A., Wieczorek B., Christensen J.R., Slifer K.J. (2010). Interdisciplinary behavioral rehabilitation of pediatric pain-associated disability: Retrospective review of an inpatient treatment protocol. J. Pediatr. Psychol..

[86] Palermo T.M., Scher M.S. (2001). Treatment of functional impairment in severe somatoform pain disorder: A case example. J. Pediatr. Psychol..

[87] Lynch M.E., Campbell F., Clark A.J., Dunbar M.J., Goldstein D., Peng P., Stinson J., Tupper H. (2008). A systematic review of the effect of waiting for treatment for chronic pain. Pain.

[88] Thompson L.A., Knapp C.A., Feeg V., Madden V.L., Shenkman E.A. (2010). Pediatricians’ management practices for chronic pain. J. Palliat. Med..

[89] Simons L.E., Smith A., Ibagon C., Coakley R., Logan D.E., Schechter N., Borsook D., Hill J.C. (2015). Pediatric pain screening tool: Rapid identification of risk in youth with pain complaints. Pain.

[90] Society A.P. Pediatric Chronic Pain Programs by State/Province. http://americanpainsociety.org/uploads/get-involved/PainClinicList_12_2015.pdf.

[91] Cormier S., Lavigne G.L., Choiniere M., Rainville P. (2016). Expectations predict chronic pain treatment outcomes. Pain.

[92] Lynch-Jordan A.M., Sil S., Peugh J., Cunningham N., Kashikar-Zuck S., Goldschneider K.R. (2014). Differential changes in functional disability and pain intensity over the course of psychological treatment for children with chronic pain. Pain.

[93] Sherry D.D., Wallace C.A., Kelley C., Kidder M., Sapp L. (1999). Short- and long-term outcomes of children with complex regional pain syndrome type I treated with exercise therapy. Clin. J. Pain.

[94] Odell S., Logan D.E. (2013). Pediatric pain management: The multidisciplinary approach. J. Pain Res..

[95] Lee B.H., Scharff L., Sethna N.F., McCarthy C.F., Scott-Sutherland J., Shea A.M., Sullivan P., Meier P., Zurakowski D., Masek B.J. (2002). Physical therapy and cognitive-behavioral treatment for complex regional pain syndromes. J. Pediatr..

[96] Wilson A.C., Palermo T.M. (2012). Physical activity and function in adolescents with chronic pain: A controlled study using actigraphy. J. Pain.

[97] Jerstad S.J., Boutelle K.N., Ness K.K., Stice E. (2010). Prospective reciprocal relations between physical activity and depression in female adolescents. J. Consult. Clin. Psychol..

[98] Bowering K.J., O’Connell N.E., Tabor A., Catley M.J., Leake H.B., Moseley G.L., Stanton T.R. (2013). The effects of graded motor imagery and its components on chronic pain: A systematic review and meta-analysis. J. Pain.

[99] Bussing A., Ostermann T., Ludtke R., Michalsen A. (2012). Effects of yoga interventions on pain and pain-associated disability: A meta-analysis. J. Pain.

[100] Evans S., Moieni M., Taub R., Subramanian S.K., Tsao J.C., Sternlieb B., Zeltzer L.K. (2010). Iyengar Yoga for young adults with rheumatoid arthritis: Results from a mixed-methods pilot study. J. Pain Symptom Manag..

[101] Vas J., Santos-Rey K., Navarro-Pablo R., Modesto M., Aguilar I., Campos M.A., Aguilar-Velasco J.F., Romero M., Parraga P., Hervas V. (2016). Acupuncture for fibromyalgia in primary care: A randomised controlled trial. Acupunct. Med..

[102] Verkamp E.K., Flowers S.R., Lynch-Jordan A.M., Taylor J., Ting T.V., Kashikar-Zuck S. (2013). A survey of conventional and complementary therapies used by youth with juvenile-onset fibromyalgia. Pain Manag. Nurs..

[103] Friedrichsdorf S., Kuttner L., Westendorp K., McCarty R. (2010). Integrative Pediatric Palliative Care.

[104] Kuttner L., Friedrichsdorf S.J. (2013). Hypnosis and palliative care. Therapeutic Hypnosis with Children and Adolescents.

[105] Hunt K., Ernst E. (2011). The evidence-base for complementary medicine in children: A critical overview of systematic reviews. Arch. Dis. Child..

[106] Evans S., Tsao J.C., Zeltzer L.K. (2008). Complementary and alternative medicine for acute procedural pain in children. Altern. Ther. Health Med..

[107] Richardson J., Smith J.E., McCall G., Pilkington K. (2006). Hypnosis for procedure-related pain and distress in pediatric cancer patients: A systematic review of effectiveness and methodology related to hypnosis interventions. J. Pain Symptom Manag..

[108] Hemington K.S., Coulombe M.A. (2015). The periaqueductal gray and descending pain modulation: Why should we study them and what role do they play in chronic pain?. J. Neurophysiol..

[109] Valet M., Sprenger T., Boecker H., Willoch F., Rummeny E., Conrad B., Erhard P., Tolle T.R. (2004). Distraction modulates connectivity of the cingulo-frontal cortex and the midbrain during pain—An FMRI analysis. Pain.

[110] Tracey I., Ploghaus A., Gati J.S., Clare S., Smith S., Menon R.S., Matthews P.M. (2002). Imaging attentional modulation of pain in the periaqueductal gray in humans. J. Neurosci..

[111] Derbyshire S.W., Osborn J. (2007). Modeling pain circuits: How imaging may modify perception. Neuroimaging Clin. N. Am..

[112] Bingel U., Wanigasekera V., Wiech K., Ni Mhuircheartaigh R., Lee M.C., Ploner M., Tracey I. (2011). The effect of treatment expectation on drug efficacy: Imaging the analgesic benefit of the opioid remifentanil. Sci. Transl. Med..

[113] Yu R., Gollub R.L., Spaeth R., Napadow V., Wasan A., Kong J. (2014). Disrupted functional connectivity of the periaqueductal gray in chronic low back pain. Neuroimage Clin..

[114] Giordano J. (2005). The neurobiology of nociceptive and anti-nociceptive systems. Pain Phys..

[115] Tegethoff M., Belardi A., Stalujanis E., Meinlschmidt G. (2015). Comorbidity of mental disorders and chronic pain: Chronology of onset in adolescents of a national representative cohort. J. Pain.

[116] Cohen L.L., Vowles K.E., Eccleston C. (2010). The impact of adolescent chronic pain on functioning: Disentangling the complex role of anxiety. J. Pain.

[117] Palermo T.M., Eccleston C., Lewandowski A.S., Williams A.C., Morley S. (2010). Randomized controlled trials of psychological therapies for management of chronic pain in children and adolescents: An updated meta-analytic review. Pain.

[118] Eccleston C., Palermo T.M., Williams A.C., Lewandowski Holley A., Morley S., Fisher E., Law E. (2014). Psychological therapies for the management of chronic and recurrent pain in children and adolescents. Cochrane Database Syst. Rev..

[119] Kashikar-Zuck S., Sil S., Lynch-Jordan A.M., Ting T.V., Peugh J., Schikler K.N., Hashkes P.J., Arnold L.M., Passo M., Richards-Mauze M.M. (2013). Changes in pain coping, catastrophizing, and coping efficacy after cognitive-behavioral therapy in children and adolescents with juvenile fibromyalgia. J. Pain.

[120] Seminowicz D.A., Shpaner M., Keaser M.L., Krauthamer G.M., Mantegna J., Dumas J.A., Newhouse P.A., Filippi C.G., Keefe F.J., Naylor M.R. (2013). Cognitive-behavioral therapy increases prefrontal cortex gray matter in patients with chronic pain. J. Pain.

[121] Forgeron P.A., McGrath P., Stevens B., Evans J., Dick B., Finley G.A., Carlson T. (2011). Social information processing in adolescents with chronic pain: My friends don’t really understand me. Pain.

[122] Palermo T.M., Wilson A.C., Lewandowski A.S., Toliver-Sokol M., Murray C.B. (2011). Behavioral and psychosocial factors associated with insomnia in adolescents with chronic pain. Pain.

[123] Palermo T.M., Law E., Churchill S.S., Walker A. (2012). Longitudinal course and impact of insomnia symptoms in adolescents with and without chronic pain. J. Pain.

[124] Letter H.H. Blue Light Has a Dark Side. http://www.health.harvard.edu/staying-healthy/blue-light-has-a-dark-side.

[125] Gooley J.J., Chamberlain K., Smith K.A., Khalsa S.B., Rajaratnam S.M., van Reen E., Zeitzer J.M., Czeisler C.A., Lockley S.W. (2011). Exposure to room light before bedtime suppresses melatonin onset and shortens melatonin duration in humans. J. Clin. Endocrinol. Metab..

[126] Logan D.E., Simons L.E., Carpino E.A. (2012). Too sick for school? Parent influences on school functioning among children with chronic pain. Pain.

[127] Bernstein G.A., Hektner J.M., Borchardt C.M., McMillan M.H. (2001). Treatment of school refusal: One-year follow-up. J. Am. Aca. Child Adolesc. Psychiatry.

[128] King N.J., Bernstein G.A. (2001). School refusal in children and adolescents: A review of the past 10 years. J. Am. Aca. Child Adolesc. Psychiatry.

[129] Evans L.D. (2000). Functional school refusal subtypes: Anxiety, avoidance, malingering. Psychol. Sch..

[130] Lewandowski A.S., Palermo T.M., Stinson J., Handley S., Chambers C.T. (2010). Systematic review of family functioning in families of children and adolescents with chronic pain. J. Pain.

[131] Liossi C., White P., Croome N., Hatira P. (2012). Pain-related bias in the classification of emotionally ambiguous facial expressions in mothers of children with chronic abdominal pain. Pain.

[132] Higgins K.S., Birnie K.A., Chambers C.T., Wilson A.C., Caes L., Clark A.J., Lynch M., Stinson J., Campbell-Yeo M. (2015). Offspring of parents with chronic pain: A systematic review and meta-analysis of pain, health, psychological, and family outcomes. Pain.

[133] Kaasboll J., Lydersen S., Indredavik M.S. (2012). Psychological symptoms in children of parents with chronic pain-the hunt study. Pain.

[134] Peloso P.M. (2014). Faster, higher, stronger: To the gold medal podium?. Pain.

[135] Moore R.A., Derry S., Straube S., Ireson-Paine J., Wiffen P.J. (2014). Faster, higher, stronger? Evidence for formulation and efficacy for ibuprofen in acute pain. Pain.

[136] Dowell D., Haegerich T.M., Chou R. (2016). Cdc guideline for prescribing opioids for chronic pain—United States, 2016. MMWR Recomm. Rep..

[137] Schechter N.L., Walco G.A. (2016). The potential impact on children of the CDC guideline for prescribing opioids for chronic pain: Above all, do no harm. JAMA Pediatr..

[138] Friedrichsdorf S.J., Nugent A.P. (2013). Management of neuropathic pain in children with cancer. Curr. Opin. Support. Palliat. Care.

[139] Vidor L.P., Torres I.L., Custodio de Souza I.C., Fregni F., Caumo W. (2013). Analgesic and sedative effects of melatonin in temporomandibular disorders: A double-blind, randomized, parallel-group, placebo-controlled study. J. Pain Symptom Manag..

[140] Schwertner A., Conceicao Dos Santos C.C., Costa G.D., Deitos A., de Souza A., de Souza I.C., Torres I.L., da Cunha Filho J.S., Caumo W. (2013). Efficacy of melatonin in the treatment of endometriosis: A phase II, randomized, double-blind, placebo-controlled trial. Pain.

[141] Finnerup N.B., Attal N., Haroutounian S., McNicol E., Baron R., Dworkin R.H., Gilron I., Haanpaa M., Hansson P., Jensen T.S. (2015). Pharmacotherapy for neuropathic pain in adults: A systematic review and meta-analysis. Lancet Neurol..

[142] Hauer J.M., Solodiuk J.C. (2015). Gabapentin for management of recurrent pain in 22 nonverbal children with severe neurological impairment: A retrospective analysis. J. Palliat. Med..

[143] Edwards L., DeMeo S., Hornik C.D., Cotten C.M., Smith P.B., Pizoli C., Hauer J.M., Bidegain M. (2016). Gabapentin use in the neonatal intensive care unit. J. Pediatr..

[144] Derry S., Wiffen P.J., Moore R.A., Quinlan J. (2014). Topical lidocaine for neuropathic pain in adults. Cochrane Database Syst. Rev..

[145] El-Chammas K., Keyes J., Thompson N., Vijayakumar J., Becher D., Jackson J.L. (2013). Pharmacologic treatment of pediatric headaches: A meta-analysis. JAMA Pediatr..

[146] Campo J.V., Perel J., Lucas A., Bridge J., Ehmann M., Kalas C., Monk K., Axelson D., Birmaher B., Ryan N. (2004). Citalopram treatment of pediatric recurrent abdominal pain and comorbid internalizing disorders: An exploratory study. J. Am. Acad. Child. Adolesc. Psychiatry.

[147] Caperell K., Pitetti R., Cross K.P. (2013). Race and acute abdominal pain in a pediatric emergency department. Pediatrics.

[148] Friedrichsdorf S.J., Drake R., Webster L.M. (2011). Gastrointestinal symptoms. Textbook of Interdisciplinary Pediatric Palliative Care.

[149] Friedrichsdorf S.J., Farquhar-Smith P., Wigmore T. (2011). Cancer pain management in children. Anaesthesia, Intensive Care, and Pain Management for the Cancer Patient.

[150] Michelson J.D., Addante R.A., Charlson M.D. (2013). Multimodal analgesia therapy reduces length of hospitalization in patients undergoing fusions of the ankle and hindfoot. Foot Ankle Int..

[151] Giordano J. (2009). The neuroscience of pain, and the neuroethics of pain care. Neuroethics.

[152] Giordano J. (2004). Pain research: Can paradigmatic revision bridge the demands of medicine, scientific philosophy and ethics?. Pain Phys..

[153] Jorgenson L.A., Newsome W.T., Anderson D.J., Bargmann C.I., Brown E.N., Deisseroth K., Donoghue J.P., Hudson K.L., Ling G.S., MacLeish P.R. (2015). The brain initiative: Developing technology to catalyse neuroscience discovery. Philos. Trans. R. Soc. Lond. Ser. B Biol. Sci..

[154] Giordano J., Schatman M.E. (2011). Pain medicine from “bench to bedside”: Bridging the disconnect(s) between research and clinical care. J. Humanit. Sci. Healthc..

[155] Giordano J. (2009). Pain: Mind, Meaning, and Medicine.

[156] Giordano J. (2008). Maldynia: Chronic pain as illness, and the need for complementarity in pain care. Forsch. Komplement..

[157] Giordano J. (2013). Pain and suffering: Körper and leib, and the telos of pain care. Philos. Psychiatry Psychol..

[158] Giordano J., Benedikter R., Boswell M.V. (2010). Pain medicine, biotechnology and market effects: Tools, tekne and moral responsibility. J. Ethics Biol. Eng. Med..

[159] Desai K., Daughtree C., Friedrichsdorf S.J. An interdisciplinary pain clinic: A year in review (poster). Proceedings of the 10th International Forum on Pediatric Pain (IFPP), White Point Beach Resort.

[160] Friedrichsdorf S.J., Postier A., Eull D., Gilbert M., Desai K., Gibbon C., Flood A. (2016). Interdisciplinary pediatric outpatient pain clinic: Improvement in pain, function and predictors of successful graduation.

[161] “Little Stars—Treating Chronic Pain in Children”. https://www.youtube.com/watch?t=13&v=Bb1fHxfjdWI.

[162] Pediatric Pain Clinics in USA and Canada (American Pain Society). http://americanpainsociety.org/uploads/get-involved/PainClinicList_12_2015.pdf.

[163] Pain Bytes (Australia). http://www.aci.health.nsw.gov.au/chronic-pain/painbytes.

[164] Persistent (Chronic) Pain 5-min video[164]. https://www.youtube.com/watch?v=RWMKucuejIs.

[165] Kiran Stordalen and Horst Rechelbacher Pediatric Pain, Palliative and Integrative Medicine Clinic Tour. https://vimeo.com/122654881.

[166] Elliot Krane (TED-Talk) The mystery of chronic pain. https://www.youtube.com/watch?v=J6--CMhcCfQ.

[167] The Department of Pain Medicine, Palliative Care, and Integrative Medicine, Children’s Hospitals and Clinics of Minnesota. https://www.childrensmn.org/painpalliativeintegrativemed.

